# Big Data Analysis of Sports and Physical Activities among Korean Adolescents

**DOI:** 10.3390/ijerph17155577

**Published:** 2020-08-02

**Authors:** Sung-Un Park, Hyunkyun Ahn, Dong-Kyu Kim, Wi-Young So

**Affiliations:** 1 Department of Sport & Leisure Studies, College of Arts & Physical Education, Shingyeong University, Hwaseong-si 18274, Korea; psu@sgu.ac.kr; 2 Department of Sport & Leisure Studies, Division of Arts & Health, Myongji College, Seoul 03656, Korea; ahnhk@mjc.ac.kr; 3 Department of Sport Management, Graduate School of Technology Management, Kyung Hee University, Yongin-si 17104, Korea; 4 Sports and Health Care Major, College of Humanities and Arts, Korea National University of Transportation, Chungju-si 27469, Korea

**Keywords:** Korean adolescents, sports, physical activities

## Abstract

The Korean government (Ministry of Culture, Sports and Tourism, Ministry of Health and Welfare, and Ministry of Education) has framed policies and conducted many projects to encourage adolescents to be more physically active. Despite these efforts, the participation rate of physical activity in Korean adolescents keeps decreasing. Thus, the purpose of this study was to analyze the perception of sports and physical activity in Korean adolescents through big data analysis of the last 10 years and to provide research data and statistical direction with regard to sports and physical activity participation in Korean adolescents. For data collection, data from 1 January 2010 to 31 December 2019 were collected from Naver (NAVER Corp., Seongnam, Korea), Daum (Kakao Corp., Jeju, Korea), and Google (Alphabet Inc., Mountain View, CA, USA), which are the most widely used search engines in Korea, using TEXTOM 4.0 (The Imc Inc., Daegu, Korea), a big data collection and analysis solution. Keywords such as “adolescent + sports + physical activity” were used. TEXTOM 4.0 can generate various collection lists at once using keywords. Collected data were processed through text mining (frequency analysis, term frequency–inverse document frequency analysis) and social network analysis (SNA) (degree centrality, convergence of iterated correlations analysis) by using TEXTOM 4.0 and UCINET 6 social network analysis software (Analytic Technologies Corp., Lexington, KY, USA). A total of 9278 big data (10.36 MB) were analyzed. Frequency analysis of the top 50 terms through text mining showed exercise (872), mind (851), health (824), program (782), and burden (744) in a descending order. Term frequency–inverse document frequency analysis revealed exercise (2108.070), health (1961.843), program (1928.765), mind (1861.837), and burden (1722.687) in a descending order. SNA showed that the terms with the greatest degree of centrality were exercise (0.02857), program (0.02406), mind (0.02079), health (0.02062), and activity (0.01872) in a descending order. Convergence of the iterated correlations analysis indicated five clusters: exercise and health, child to adult, sociocultural development, therapy, and program. However, female gender, sports for all, stress, and wholesome did not have a high enough correlation to form one cluster. Thus, this study provides basic data and statistical direction to increase the rate of physical activity participation in Korean adolescents by drawing significant implications based on terms and clusters through bid data analysis.

## 1. Introduction

The 2018 Korea Student Health Examination reported that the rate of obesity in Korean adolescents increased from 21.2% in 2014 to 25.0% in 2018, representing an increase of 3.8 percentage points in three years [1]. Furthermore, a recent report indicated that the percentage of students who engaged in the recommended levels of exercise (strenuous exercise three or more days per week) was 59.25% among elementary school students, 35.08% among middle school students, and 23.60% among high school students, suggesting a declining trend in exercise with age among children and adolescents [1]. The increase in the rate of obesity among adolescents is troubling because research indicates that 80% of obese adolescents become obese adults [2]. Therefore, adolescence is a crucial period for developing healthy habits [3,4,5]. However, as the statistics above suggest, less than 25% of Korean adolescents engage in the recommended levels of exercise by the time they reach high school.

Big data refers to large-scale data that cannot be stored, managed, or analyzed using traditional database software [6]. Big data is distinct from standard data in terms of volume, velocity, and variety [7]. Currently, an explosive increase in the amount of big data collected is taking place [8,9]. Big data has become an important part of research due to a significant increase in unstructured data recently [10]. Research based on big data analysis reveals interesting insights on consumer perception, choice, emotion, and personal intention to act. It can also identify market perception, trends, and make predictions through the analysis of patterns [11]. However, big data must be handled by a reliable system with a formal data policy for usage and storage [12,13] that is capable of conducting large data calculations. Big data is particularly useful because new insights or values that cannot be derived from small amounts of data can be extracted and used to initiate important changes in various areas including market, corporate, civic, and governmental relationships [14].

Korea currently offers favorable conditions for big data to flourish by virtue of its globally superior network infrastructures and the immense amount of data consequently produced [13,15]. While big data is certainly a hot topic and a growing development target, most governments and companies are still not actively applying data analytics [13]. An examination of the obesity rate of Korean adolescents combined with their rate of engagement in the recommended levels of exercise reveals the need to examine their current perceptions of sports and physical activities (SPA). Big data analysis may suggest a strategic direction related to Korean adolescents’ SPA that can inform the development of interventions aimed at increasing the rate of engagement in the recommended levels of exercise, and in turn decreasing the obesity rate among Korean adolescents. Therefore, this study aims to collect and analyze big data [16] to examine Korean adolescents’ perceptions of SPA.

## 2. Materials and Methods

### 2.1. Data Collection

This study was approved by the Institutional Review Board of Kyung Hee University, Gyeonggi, Korea (No. KHGIRB-20-096). Data were searched from 1 January 2010 to 31 December 2019 to be included in the analysis. For data collection, the TEXTOM 4.0 big data analysis solution (The Imc Inc., Daegu, Korea), a web crawling program, was used to collect the unstructured text on webpages, blogs, and news articles provided by Naver [17], Daum, and Google [18]. The terms “adolescent + sports + physical activity” were used as search keywords. TEXTOM has an adding keyword function that can collect data using keywords. Using the adding keyword function has an advantage as it can generate various collection lists at once [19]. Moreover, the keywords were searched separately (not as a phrase) in this study. Moreover, Naver, Daum, and Google were set as collection channels due to the fact that Naver, Daum, and Google showed 77, 10.8, and 1.7% of Korean Internet searches in order [20,21]. We found that Google did not display satisfying results due to a lack of Korean data even though it is a worldwide and strong search engine [20]. The information on the collected data is shown in Table 1.

### 2.2. Data Analysis

In this study, text mining and social network analysis (SNA) were performed to analyze big data on Korean adolescents’ SPA. Text mining refers to the technique of using natural language processing and data mining techniques to extract meaningful information from unstructured text data [22]. Thus, text mining is used to analyze vast amounts of text to extract patterns or relationships, discover meaningful values, and interpret them with insight [23]. Therefore, a frequency analysis and term frequency–inverse document frequency (TF–IDF) analysis were derived using text mining. Frequency analysis refers to the number of times that a word or term appears in a document, and the TF–IDF approach is commonly used to weigh each word in the text document, according to how unique it is [24]. Second, SNA is a method of quantitatively analyzing the characteristics of a social network [25] by focusing on the patterns of relations among the entities in the network (e.g., people, organizations, and states [16,26]).

Network centrality is a measure of how close each node in the network is to the center of the network [27]. There are multiple measures of network centrality, but degree centrality, the most representative of the measures, is also the most reliable and simplest [28]. Degree centrality is a measure of how many neighbors a node has; a word that has many connections to other words becomes more central, giving it a greater impact on other words and a more dominant role in the network [29,30]. Thus, degree centrality is an index of the degree to which a particular node is located toward the center of the overall network [31,32,33]. Additionally, the CONCOR (CONvergence of iterated CORrelations) analysis is the process of discovering patterns in the relationships between words, and the greater the similarity of the relationship patterns, the greater the degree of structural equivalence of the other words [30].

In this study, degree centrality and CONCOR, which are the most representative concepts in SNA, were used. TEXTOM 4.0 big data analysis solution (The Imc Inc., Daegu, Korea) and UCINET 6 social network analysis software (Analytic Technologies Corp., Lexington, KY, USA) were used to perform text mining and SNA [34].

## 3. Results

### 3.1. Results of Data Collection

In this study, texts related to the keywords “adolescent + sports + physical activity”, published on Naver, Daum, and Google between 1 January 2010 and 31 December 2019 were collected; the results are reported in Table 2. In total, 9278 data points were collected using TEXTOM 4.0 big data analysis solution and the total data volume was 10.36 MB.

### 3.2. Text Mining Analysis

First, the results of performing a frequency analysis on the top 50 terms related to Korean adolescents’ SPA are shown in Table 3. The results showed the top 25 most frequently used terms were exercise (872), mind (851), health (824), program (782), burden (744), vitamin D (737), outdoor activity (734), immunity (729), sunbathing (719), activity (633), management (538), school (520), children (488), participation (429), education (415), student (401), social (354), growth (349), mental (336), child (321), development (305), kid (279), body (273), game (260), and opportunity (258) in descending order.

Second, TF–IDF was performed to calculate how important each term was in a particular document by multiplying term frequency (TF) and inverse document frequency (IDF). TF means the frequency of a specific word in a document, DF is the frequency of a specific word in multiple documents, and IDF is the inverse of DF [19]. Thus, the TF–IDF value increases as the frequency of a word in a specific document increases and the number of documents that include the specific word decrease. The basic formula to calculate this TF–IDF value is as follows [19,35]:

TF–IDF = TF × 1/DF

As seen in Table 4, the results of the TF–IDF analysis were similar to those of the frequency analysis, with the following results in descending order: exercise (2108.070), health (1961.843), program (1928.765), mind (1861.837), burden (1722.687), vitamin D (1718.496), outdoor activity (1707.490), immunity (1702.844), sunbathing (1687.441), activity (1599.081), management (1507.636), school (1490.146), children (1431.463), participation (1255.191), education (1251.933), student (1218.513), social (1112.992), growth (1086.663), child (1068.955), mental (1056.399), development (1019.094), kid (964.428), skin (963.135), game (937.734), and body (933.512).

### 3.3. Social Network Analysis

This study was based on degree centrality, which focuses on the level of connection of one node to the others as the centrality. Furthermore, to analyze the structures of the relationships among the latent sub-clusters, CONCOR analysis was performed. First, normalized degree centrality is defined as the number of links divided by the maximum possible value [36]. Thus, the closer it is to 1, the higher the degree centrality. A higher degree centrality value was interpreted to mean that there was a significant number of links among terms and a significant impact in the network. Therefore, to test how connected the derived terms were to “adolescent + sports + physical activity”, a degree centrality analysis was performed, the results of which are shown in Table 5. The results of the degree centrality analysis were exercise (0.02857), program (0.02406), mind (0.02079), health (0.02062), activity (0.01872) management (0.01545), student (0.01525), participation (0.01491), school (0.01475), education (0.01375), children (0.01305), child (0.01184), kid (0.01094), social (0.01064), mental (0.00964), development (0.00924), person (0.00921), physical education (0.00917), growth (0.00911), physical activity (0.00857), opportunity (0.00851), body (0.00831), time (0.00831), game (0.00821), and stress (0.00807) in a decreasing order. In particular, the results of the degree centrality analysis showed higher rankings of nodes such as activity, management, student, participation, school, and education compared to the results of the frequency and TF–IDF analyses.

**Figure 1:**
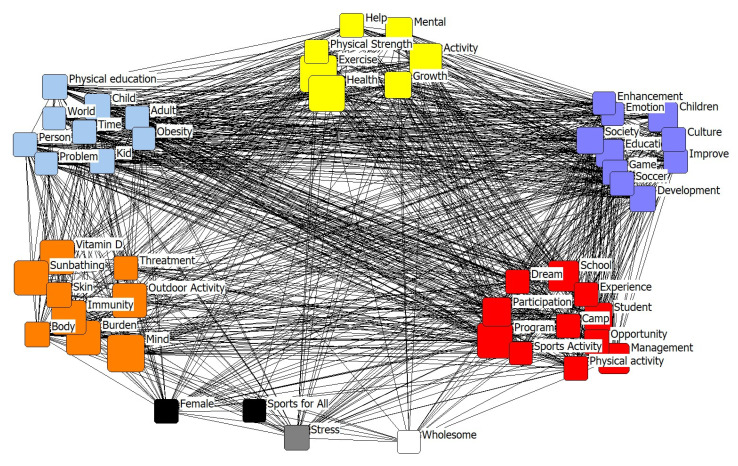
Convergence of iterated correlations analysis results. Note. yellow cluster = exercise and health; sky blue cluster = child to adult; purple cluster = sociocultural development; orange cluster = therapy; red cluster = program; black, gray, and white = could not form a cluster.

Second, a CONCOR analysis was performed to analyze the structures of the relationships among the latent sub-clusters in the network cluster. The results are shown in Figure 1 and Table 6. Based on these results, homogenous groups were identified according to relationships and correlations, resulting in five clusters. The first cluster (visualized with yellow) comprised the terms “exercise”, “health”, “activity”, “mental”, “growth”, “physical strength”, and “help”, and was categorized as “exercise and health”. The second cluster (visualized with sky-blue) comprised the terms “child”, “kid”, “physical education”, “adult”, “world”, “time”, “problem”, “person”, and “obese”, and was categorized as “child to adult.” The third cluster (visualized with purple) comprised the terms “children”, “education”, “social”, “culture”, “development”, “improvement”, “soccer”, “game”, “emotion”, and “enhancement”, and was categorized as “sociocultural development”. The fourth cluster (visualized with orange) comprised the terms “mind”, “immunity”, “vitamin D”, “outdoor activity”, “burden”, “sunbathing”, “body”, “skin”, and “treatment”, and was categorized as “therapy”. The fifth cluster (visualized with red) comprised the terms “program”, “management”, “school”, “student”, “participation”, “opportunity”, “dream”, “experience”, “physical activity”, and “sports activity”, and was categorized as the “program” cluster. However, female, sports for all, stress, and wholesome could not form a cluster (visualized with black, gray, and white).

## 4. Discussion

As a result of the frequency analysis of text-mining from 2010 to 2019, the SPA of Korean adolescents, “exercise”, “mind”, “health”, “program”, and “burden” showed high frequency. Baker et al. (2011) and Keteyian (2011) claimed that physical activities that require active performance such as sports are important for enhancing health [37,38] and that regular physical activity can improve adolescent academic achievement [7]. Additionally, regular participation in physical activity is related to child and adolescent health [39,40,41]. However, in spite of these advantages, Korean adolescents, along with those from Belgium, China, Scotland, and Taiwan, were ranked F in the overall physical activity index in the 2018 Report Card (RC), which was at the bottom of 49 countries [42]. This rank is much lower compared with Korea’s 2016 RC overall physical activity index (D−) [43]. Thus, there is a need to focus on Korean SPA continuously. In particular, the keyword “burden” was recurrent in the findings, indicating that there are practical barriers against sports and physical activities in Korean society. Furthermore, the results of the degree centrality analysis showed that the ranks of nodes such as “activity”, “management”, “student”, “participation”, and “school” were higher compared to the results of the frequency and TF–IDF analyses. This, together with the prevailing prioritization of academic achievements in Korean society, leads to the inference that there is a tendency to prioritize studies over sports and physical activities. Considering the sociocultural background in which academic achievements are more highly valued than SPA in Korea, SPA in schools should be further strengthened.

The results of the CONCOR analysis categorized the structural similarities within the network into five clusters: “exercise and health”, “child to adult”, “sociocultural development”, “therapy”, and “program”. First, in the “exercise and health” cluster, it was found that the links between exercise, health, and activity were high. This supports the findings of previous studies suggesting that sports and physical activities are important factors for adolescent growth [44] and health [37,38]. Second, in the “child to adult” cluster, the links between “child”, “kid”, and “physical education” were found to be high. It has been suggested that the interest in sports and physical activities was higher among children. In particular, as regular physical activities in adolescence can improve physical activities in adulthood [45], it is important to form good habits related to SPA in adolescence. Third, in the “sociocultural development” cluster, links between “children”, “social”, and “education” were found to be high, indicating that sociocultural background relates to Korean adolescents’ SPA. Lindquist, Reynolds, and Goran (1999) criticized the insufficient research on the impact of pervasive sociocultural factors on children’s physical activity and physical strength, despite its latent impact on various physical activities [46]. Therefore, in-depth research on the relationship between sociocultural factors and SPA of Korean adolescents is urgently needed. Fourth, in the “therapy” cluster, links between “mind”, “immunity”, and “vitamin D” were found to be high. Thus, SPA can be speculated to enhance the mental wellbeing and immune system of adolescents. Physical activities and mental health are highly related in adolescence [47], and it has been shown that regular exercise has an effect on the immune system and can even delay aging [48]. Fifth, in the “program” cluster, the links between “program”, “management”, and “school” were found to be high. Practical SPA programs that consider the age and target as well as expand the time devoted to physical education in schools and related after school sports clubs are recommended. Exercise levels during adolescence should be increased through the planning and implementation of mid- to long-term SPA at the sociocultural and national levels. Finally, it was shown that terms such as female, sports for all, stress, and wholesome had no high correlation and therefore did not form clusters. However, it seems necessary to pay attention to deduced terms.

## 5. Conclusions

In this study, big data related to Korean adolescents’ SPA between 1 January 2010 and 31 December 2019 were collected, and text mining and SNA were performed on the collected unstructured text using the TEXTOM 4.0 big data analysis solution (The Imc Inc., Daegu, Republic of Korea) and UCINET 6 social network analysis software (Analytic Technologies Corp., Lexington, KY, USA).

The total number of big data analyzed in this study was 9278 data points, and the volume was 10.36 MB. The results of the frequency analysis through text mining showed that the terms “exercise”, “mind”, “health”, “program”, “burden”, “vitamin D”, “outdoor activity”, “immunity”, “sunbathing”, and “activity” were the most frequently used words. The results of the TF–IDF analysis showed that “exercise”, “health”, “program”, “mind”, “burden”, “vitamin D”, “outdoor activity”, “immunity”, “sunbathing”, and “activity” were the most frequently used words. Through the analytic process, various nodes related to Korean adolescents’ SPA and their relative importance were identified.

Second, the results of the SNA showed that the terms with the greatest degree of centrality were “exercise”, “program”, “mind”, “health”, “activity”, “management”, “student”, “participation”, “school”, and “education”. Nodes such as “activity”, “management”, “student”, “participation”, “school”, and “education” were found to have an increased ranking in the SNA results compared to the results of the frequency analysis and TF–IDF analysis. The results of the CONCOR analysis yielded the following five clusters: exercise and health, child to adult, sociocultural development, therapy, and program. However, even though female, sports for all, stress, and wholesome could not form a cluster, circumspection is required. In conclusion, three Korean ministries such as the Ministry of Culture, Sports and Tourism, Ministry of Health and Welfare, and Ministry of Education have conducted and planned about 190 policies and projects with regard to the physical activity of children and adolescents [49]. Despite these efforts, the physical activity index of Korean adolescents is decreasing more and more [43]. Thus, this research provides specific and systematic facts about Korean adolescents’ SPA based on big data from the past 10 years. Furthermore, the participation rate in sports and physical activities among Korean adolescents may be improved if the sports and physical activity cluster is divided based on deducted cluster and problems, and improvement points of each cluster can be supplemented. With this knowledge, Korean SPA programs that consider the clusters can be developed in follow-up research based on the results of this study.

## Figures and Tables

**Table 1:** Text data collection information.

Category	Content
Collection channel	Naver, Daum, Google
Collection period	1 January 2010 to 31 December 2019
Collection tool	TEXTOM 4.0 big data analysis solution (The Imc Inc., Daegu, Korea) (http://textom.co.kr)
Analysis keyword	Adolescents, Sports, Physical activities
Analysis tool	TEXTOM 4.0 big data analysis solution (The Imc Inc., Daegu, Korea) (http://textom.co.kr), UCINET 6 social network analysis software (Analytic Technologies Corp., Lexington, KY, USA) (http://www.analytictech.com)

**Table 2:** Collection channel, number of data points, and volume.

Collection Channel	Number of Data Points	Volume
Naver	5343	2.18 MB
Daum	3401	1.19 MB
Google	534	6.99 MB
Total	9278	10.36 MB

**Table 3:** Results of the frequency analysis.

Rank	Term	Freq.	Rank	Term	Freq.
1	Exercise	872	26	Stress	257
2	Mind	851	27	Physical education	256
3	Health	824	28	Skin	251
4	Program	782	29	Adult	232
5	Burden	744	30	Improve	230
6	Vitamin D	737	31	Physical activity	229
7	Outdoor activity	734	32	Dream	228
8	Immunity	729	33	Female	227
9	Sunbathing	719	34	Experience	226
10	Activity	633	35	Soccer	221
11	Management	538	36	Physical strength	213
12	School	520	37	Person	211
13	Children	488	38	Treatment	209
14	Participation	429	39	Help	203
15	Education	415	40	Camp	197
16	Student	401	41	Culture	196
17	Society	354	42	Time	196
18	Growth	349	43	Sports activity	187
19	Mental	336	44	World	183
20	Child	321	45	Obesity	182
21	Development	305	46	Wholesome	175
22	Kid	279	47	Emotion	174
23	Body	273	48	Problem	173
24	Game	260	49	Enhancement	171
25	Opportunity	258	50	Sport for all	166

**Table 4:** Term frequency–inverse document frequency analysis results.

Rank	Term	Freq.	Rank	Term	Freq.
1	Exercise	2108.070	26	Physical education	906.315
2	Health	1961.843	27	Female	896.207
3	Program	1928.765	28	Stress	894.970
4	Mind	1861.837	29	Opportunity	878.059
5	Burden	1722.687	30	Adult	824.563
6	Vitamin D	1718.496	31	Physical activity	822.584
7	Outdoor activity	1707.490	32	Soccer	822.293
8	Immunity	1702.844	33	Treatment	820.864
9	Sunbathing	1687.441	34	Improve	819.604
10	Activity	1599.081	35	Dream	812.477
11	Management	1507.636	36	Experience	811.808
12	School	1490.146	37	Physical strength	787.896
13	Children	1431.463	38	Person	773.799
14	Participation	1255.191	39	Camp	769.779
15	Education	1251.933	40	Help	738.214
16	Student	1218.513	41	Culture	731.439
17	Society	1112.992	42	World	727.613
18	Growth	1086.663	43	Sport for all	727.328
19	Child	1068.955	44	Time	726.069
20	Mental	1056.399	45	Obesity	707.586
21	Development	1019.094	46	Sports activity	693.743
22	Kid	964.428	47	Problem	677.149
23	Skin	963.135	48	Emotion	663.428
24	Game	937.734	49	Wholesome	658.000
25	Body	933.512	50	Enhancement	655.108

**Table 5:** Results of degree centrality analysis.

Rank	Term	Freq.	Rank	Term	Freq.
1	Exercise	0.02857	26	Adult	0.008077
2	Program	0.02406	27	Treatment	0.008010
3	Mind	0.02079	28	Start	0.007877
4	Health	0.02062	29	Problem	0.007576
5	Activity	0.01872	30	Skin	0.007376
6	Management	0.01545	31	Effect	0.007309
7	Student	0.01525	32	Improve	0.007109
8	Participation	0.01491	33	Culture	0.007076
9	School	0.01475	34	Help	0.007009
10	Education	0.01375	35	Sports activity	0.006909
11	Children	0.01305	36	Experience	0.006909
12	Child	0.01184	37	Physical strength	0.006809
13	Kid	0.01094	38	Soccer	0.006742
14	Society	0.01064	39	Stability	0.006508
15	Mental	0.00964	40	Method	0.006441
16	Development	0.00924	41	Increase	0.006308
17	Person	0.00921	42	Camp	0.006275
18	Physical education	0.00917	43	Perform	0.006041
19	Growth	0.00911	44	Practice	0.006041
20	Physical activity	0.00857	45	Obesity	0.006041
21	Opportunity	0.00851	46	Think	0.005874
22	Body	0.00831	47	Dream	0.005741
23	Time	0.00831	48	Athlete	0.005741
24	Game	0.00821	49	Prevent	0.005674
25	Stress	0.00807	50	Develop	0.005640

**Table 6:** Results of the convergence of iterated correlations analysis.

	Cluster	Term
1	Exercise and health	Exercise, health, activity, mental, growth, physical strength, help
2	Child to adult	Child, kid, physical education, adult, world, time, problem, person, obese
3	Sociocultural development	Children, education, social, culture, development, improvement, soccer, game, emotion, enhancement
4	Therapy	Mind, immunity, vitamin D, outdoor activity, burden, sunbathing, body, skin
5	Program	Program, management, school, student, participation, opportunity, dream, experience, physical activity, sports activity
